# Challenges associated with disclosing results from whole genome sequencing to diagnose paediatric rare diseases: Analysis of parent-clinician interactions

**DOI:** 10.1136/jmg-2025-111137

**Published:** 2026-02-20

**Authors:** Holly Ellard, Jhumana Ali, Phoebe Buxton, Myra Bluebond-Langner, Celine Lewis

**Affiliations:** 1 Population, Policy and Practice Department, UCL Great Ormond Street Institute of Child Health, London, UK; 2 Division of Psychology and Language Sciences, University College London, London, UK; 3 School of Medicine, Cardiff University, Cardiff, Wales, UK; 4 Moorfields Eye Hospital NHS Foundation Trust, London, UK; 5 Louis Dundas Centre for Palliative Care for Children and Young People, UCL Great Ormond Street Institute of Child Health, London, UK; 6 Department of Sociology, Anthropology and Criminal Justice, Rutgers University, Camden, New Jersey, USA

**Keywords:** genomic sequencing, rare disease, Genomic Medicine Service, return of results

## Abstract

**Background:**

Whole genome sequencing (WGS) has recently been introduced as a diagnostic test for patients with particular rare diseases in the NHS in England. Little is known about the process of communicating results from WGS to families in practice.

**Methods:**

We audio-recorded clinicians and parents discussing the results of WGS for their child’s rare disease diagnosis as part of a larger mixed methods evaluation of the implementation of the NHS Genomic Medicine Service during its early years.

**Results:**

Ten consultations were audio-recorded across four NHS Trusts. Clinical indications for WGS were related to neurological and developmental disorders. Seven parents received a genetic diagnosis for their child’s condition, two received a variant of uncertain significance, and one received a no primary finding result. One parent also received an incidental finding for their child. Challenges in discussing results included: 1. explaining a diagnosis when the genotype was established before detailed phenotyping, 2. navigating follow-up for an adult-onset condition identified in childhood, 3. disclosing an unexpected diagnosis for a parent from trio testing, and 4. conveying a diagnosis with an uncertain prognosis.

**Conclusion:**

This study illustrates some of the issues that can arise from unexpected and uncertain information when returning results from broad-scope genomic testing for paediatric neurological and developmental disorders. Further study of actual interactions between clinicians and families discussing results from WGS across different specialities and conditions is needed to inform guidance on communication of results within this rapidly evolving area of medicine.

## Introduction

Childhood rare diseases are predominantly genetic in origin, many of which are complex, multisystemic, and severe. A timely genetic diagnosis is key to unlocking access to specialist care and treatment, improving social and educational support, informing prognosis, facilitating reproductive decision-making, and enabling connections with other affected families [1]. However, traditional approaches to genetic testing to ascertain the cause of genetically and phenotypically heterogenous conditions can be challenging and result in an arduous ‘diagnostic odyssey’ for families [2].

A faster route to a genetic diagnosis can be achieved by investigating thousands of genes simultaneously through a large virtual gene panel (limiting analysis to genes linked to the patient’s presentation) applied after WGS. For example, neurodevelopmental disorders such as intellectual disability have a range of overlapping phenotypes and genetic aetiologies making them difficult to diagnose through traditional testing, whereas diagnostic yields of up to 55% have been obtained through WGS [3,4]. In 2018 in the NHS in England, the Genomic Medicine Service (GMS) was launched to integrate WGS into routine diagnostic testing for certain rare diseases, with over 100,000 genomes having now been sequenced [5]. The ‘National Genomic Test Directory’ specifies genomic tests available in the NHS in England, including clinical indications and eligibility for WGS [6]. Consent to WGS broadly covers potential outcomes of testing – a diagnosis, variant of uncertain significance (VUS) (a variant with insufficient evidence to conclude its role in disease), or no primary finding; the possibility of unexpected or uncertain results; implications for patients and relatives; and data governance [7,8].

Compared to more targeted approaches to genetic testing, the large volume of data generated by WGS increases the likelihood of an unexpected or uncertain result [9,10], such as an incidental finding (a variant that causes a health problem unrelated to the reason for testing); a VUS; or a newly discovered or ultra-rare condition for which knowledge about disease progression and clinical management is still evolving. Moreover, while WGS for previously intractable heterogenous conditions (such as neurodevelopmental disorders) may lead to a genetic diagnosis, variability in penetrance and expressivity of some of these conditions may leave parents no closer to answers around prognosis.

On interview, healthcare professionals report difficulties in delivering results of genomic sequencing [11,12], particularly when contending with inflated patient expectations about the likelihood or actionability of a diagnosis.

Studies of the impact of receiving a genomic result have indicated that patients/parents experience minimal adverse psychological effects and decisional regret from sequencing [13–16]. That said, individual experiences varied and negative responses often related to unrealised expectations. There is less research on how genomic results are presented and discussed with families.

Process studies have been instrumental in uncovering the content, behaviours, and activities that underpin clinical genetics consultations and are a necessary part of evaluating a service where the primary form of intervention is a process of communication [17,18]. Few process studies have been conducted since the rise of next generation sequencing technologies in clinical practice; studies have focused on how uncertainty is navigated interactionally during disclosure of results from mostly exome sequencing [19–21]. If, and how, other aspects of communication have responded to the shift from genetic to genomic practice remains unclear.

The communication that happens during consultations to return results from WGS in the NHS GMS has yet to be investigated. As the UK government continues its mission to bring genomics into mainstream medicine, and traditional models of service delivery within clinical genetics are called into question, there is a pressing need to open this black box [22,23]. This is the first study to examine what is said and done in interactions between clinicians and parents receiving WGS results for their child through the GMS. The purpose of this article is to explore some of the challenges in consultations and consider ways to address them in the context of the interactions.

## Methods

This study is part of a mixed methods evaluation of the implementation of the NHS GMS [24], contributing to the part of the evaluation that examined consent and return of results processes [8].

### Recruitment, enrolment and data collection

A clinician from the genetics department (acting as a local principal investigator) at each of the seven NHS Trusts involved in the mixed methods evaluation was approached with information about the Return of Results study and asked to distribute the information to colleagues. Clinicians that expressed interest in taking part (which could include the local principal investigators initially approached) were invited to complete a consent document and participant demographic form. Clinicians were asked to notify the study team of upcoming consultations for returning WGS results to parents/carers of a child with a rare condition that they felt were suitable for the researcher (CL or HE) to observe. Clinicians were offered the option to audio-record the consultation themselves if circumstances precluded the observer being present (e.g. scheduling issues).

Parents were eligible to participate if their child was under 16 years old; had capacity; were able to read information materials; and were having the discussion in English or with a professional translator present. Parents were approached by the clinician or researcher prior to the consultation with information about the study and asked if they were interested in taking part. Those interested were asked to complete a consent document and participant demographic form. Where possible, unless the child was too young or their capacity was impaired, the child was also given the opportunity to assent to take part.

Consultations took place between August 2022 – March 2025. Field notes were kept capturing context and non-verbal behaviour.

### Data analysis

Audio-recordings were transcribed verbatim by an external transcriptionist. Pseudonyms replaced participants’ names. Analysis was an iterative process. Initially, the researchers (HE, CL, MBL, AJ, PB) familiarised themselves with the transcripts and met to reflect upon what they perceived to be relevant to the study aims and develop a codebook containing deductive (including terms from previous studies) and inductive codes. The codebook was piloted with three transcripts. The researchers met to resolve issues and agree upon a final codebook to apply to the remaining transcripts and repeat on the original three. Coding was completed by a single researcher (HE) using NVivo (version 14.23.1). Coded data was summarised in a matrix of cases against codes, drawing on methods used in framework analysis [25]. The researchers met throughout analysis to review findings, discuss interpretations, and consider aspects of consultations that warranted further consideration in light of the challenges they generated for practice.

## Results

### Overview of consultations

Ten consultations were audio-recorded across four NHS Trusts (Table 1). Consultations were led by clinical geneticists (8/10), a genetic counsellor (1/10) or a paediatric neurologist (1/10), and attended by mothers (10/10), fathers (3/10), and the paediatric patient (7/10). Indications for testing were NDDs.

Notable challenges in interactions (described below) included: 1. explaining a diagnosis when the genotype was established before detailed phenotyping, 2. navigating follow-up for an adult-onset condition identified in childhood, 3. disclosing an unexpected diagnosis for a parent from trio testing, and 4. conveying a diagnosis with an uncertain prognosis.

#### 1 Explaining a diagnosis when the genotype was established before detailed phenotyping

**Figure 1:**
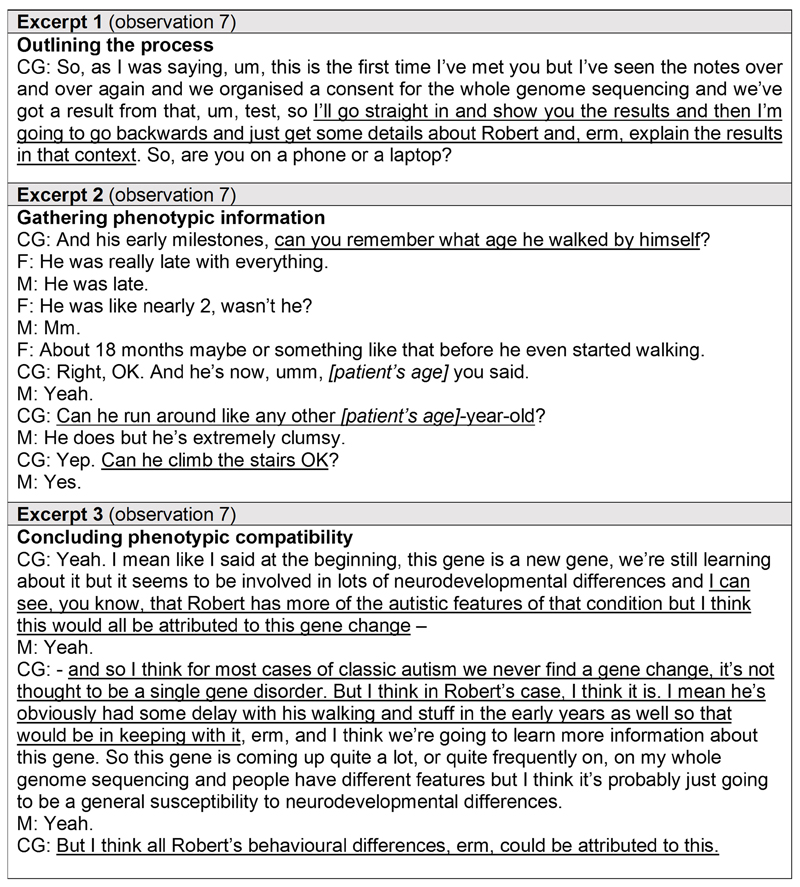
Excerpts from a verbatim transcript of a whole genome sequencing result disclosure consultation demonstrating the challenge of explaining a diagnosis when the genotype was established before detailed phenotyping. Excerpts from a verbatim transcript of a video consultation between a clinical geneticist and parents receiving a diagnosis of TBL1XR1-related disorder for their child from whole genome sequencing, illustrating an example of fitting the child’s genotype to their phenotype. Key messages are underlined. Pseudonyms are in place of participants’ real names. *CG = clinical geneticist; M = mother; F = father*.

In several cases, disclosing the results of WGS involved ‘fitting’ (judging the compatibility of) the diagnosis with the child’s clinical and developmental history. Specifically, there was an exchange of information between clinicians (as holders of the genotypic information) and parents (as holders of the phenotypic information), where they constructed in real time the genomic result in the context of the child’s phenotype. For example, the excerpts in Figure 1 taken from a consultation between a clinical geneticist and parents receiving a diagnosis of TBL1XR1-related disorder for their child illustrate an occasion when the clinician shaped the consultation around the process of gathering phenotypic information to compare with the genetic diagnosis.

The clinician began the consultation by setting out their plans to first gather details about the child, stating explicitly that the purpose of this was to contextualise the genetic diagnosis (Figure 1: Excerpt 1). After giving parents a summary of the result – briefly, a *de novo* change in the newly discovered TBL1XR1 gene that causes non-specific developmental and behavioural differences – the clinician asked a series of questions about the child’s clinical and development history (Figure 1: Excerpt 2). Once phenotypic information had been collated in this way, the clinician related that information back to the genetic diagnosis in hand, making a judgement about the fit between the genotype and phenotype (Figure 1: Excerpt 3). The clinician concluded that what they had been told about the child’s developmental delay, behavioural differences, and autism was consistent with TBL1XR1-related disorder.

#### 2 Navigating follow-up for an adult-onset condition identified in childhood

**Figure 2:**
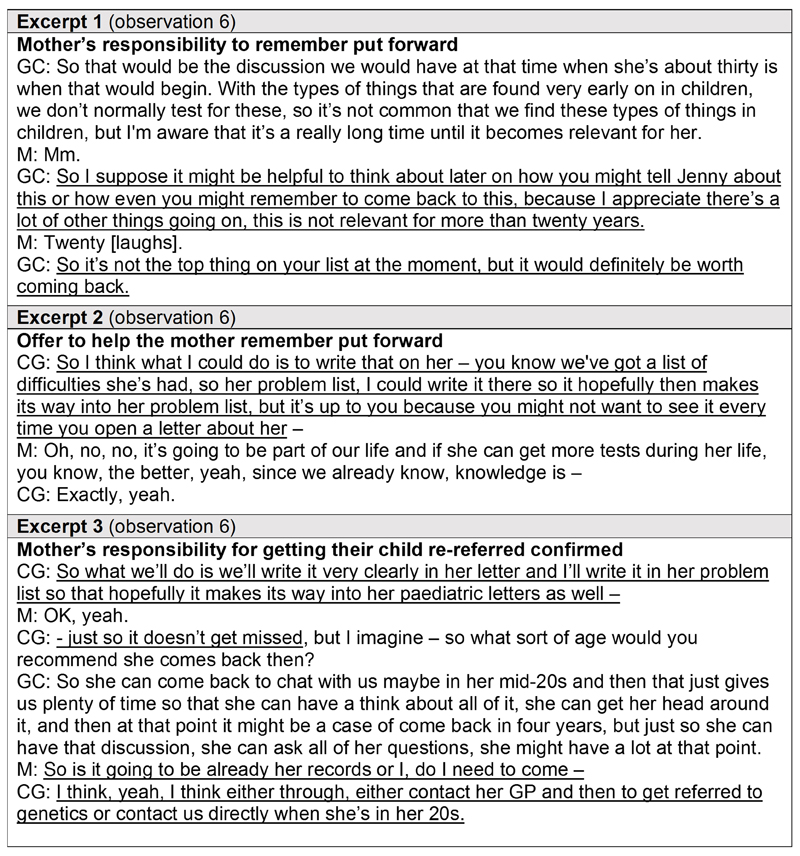
Excerpts from a verbatim transcript of a whole genome sequencing result disclosure consultation demonstrating the challenge of navigating follow-up for an adult-onset condition identified in childhood. Excerpts from a verbatim transcript of a face-to-face consultation between a clinical geneticist, genetic counsellor, and mother receiving their child’s results from diagnostic whole genome sequencing, illustrating the negotiation of responsibility for following up an adult-onset condition identified incidentally during childhood. Pseudonyms are in place of participants’ real names. Key messages are underlined. *CG = clinical geneticist; GC = genetic counsellor; M = mother*.

In one consultation, an incidental finding of hereditary breast and ovarian cancer (HBOC) – an inherited predisposition to specific adult-onset cancers – was identified in the child and discussed in addition to their main VUS finding. Clinical management for HBOC (such as screening or preventative surgeries) would not be relevant for that child until adulthood. The excerpts in Figure 2 illustrate the negotiation of onus for follow-up amongst those present at the face-to-face consultation.

Initially, the genetic counsellor implied that the mother would be responsible for re-engaging with clinical genetics and prompted her to start thinking about how she would remember to do so (Figure 2: Excerpt 1). After further discussion about the risk of cancers related to HBOC, which was upsetting for the mother, the clinical geneticist proposed adding HBOC to the child’s problem list on their health record so it would appear on their patient letters as a memory aid. However, they invited the mother to reject this idea if the emotional burden of being reminded about HBOC with every letter would be too much (Figure 2: Excerpt 2). With reassurance that the mother had accepted HBOC was now part of their family’s life, the clinical geneticist confirmed they would document this in the child’s problem list to avoid follow-up getting missed (Figure 2: Excerpt 3).

The mother then sought to clarify whether that alleviated her duty to remember to re-engage with the service. The clinical geneticist confirmed that it was still the mother’s responsibility to get re-referred once their child was in their twenties via the child’s general practitioner (GP) or contacting clinical genetics (Figure 2: Excerpt 3).

#### 3 Disclosing an unexpected diagnosis for a parent from trio testing

**Figure 3:**
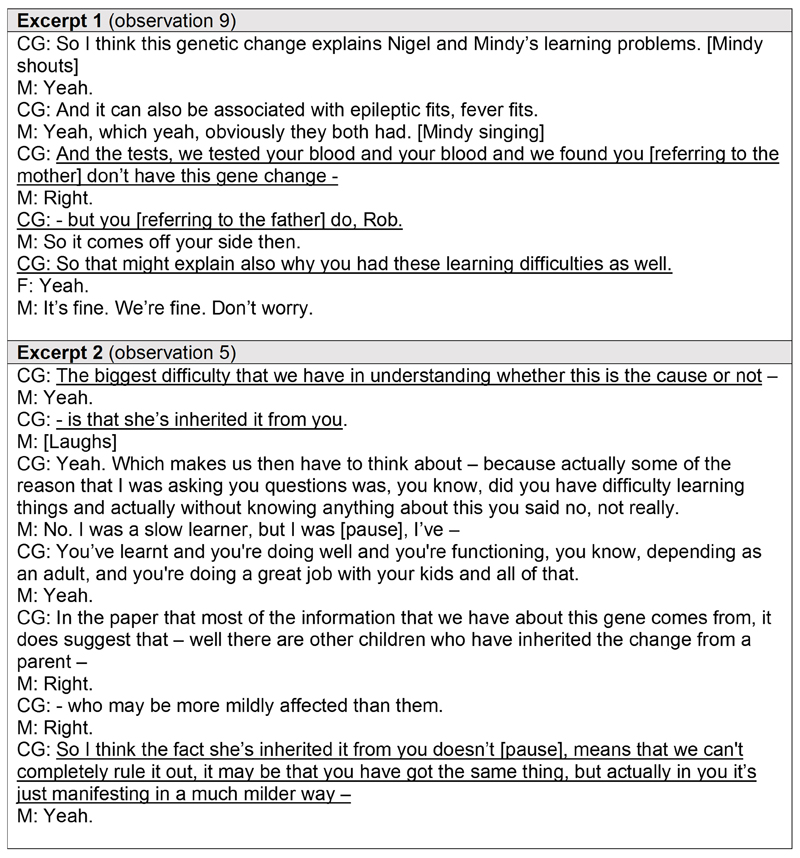
Excerpts from verbatim transcripts of whole genome sequencing result disclosure consultations demonstrating the challenge of disclosing an unexpected diagnosis for a parent from trio testing. Excerpts from verbatim transcripts of face-to-face consultations between clinicians and parents discussing the results of whole genome sequencing for their child’s rare disease diagnosis, illustrating scenarios when a parent received an unexpected (possible) diagnosis from trio testing. Pseudonyms are in place of participants’ real names. Key messages are underlined. *CG = clinical geneticist; M = mother; F = father*.

On two occasions in this study, trio testing (sequencing a child and both parents) led to a parent receiving (possible) unexpected diagnostic information about themselves. Trio testing does not intend to uncover diagnostic information about parents since the virtual panel of genes applied to sequence data is associated with conditions they are not considered to have. However, in the cases presented here, further discussion about a parent’s learning and development history *after* testing led to a re-evaluation of their disease status in light of their genotype. For example, in a face-to-face consultation between a clinical geneticist, genetic counsellor, and parents of siblings receiving a diagnosis of SEN8A-related disorder, the clinical geneticist asked the father a series of questions about his health and experiences at school before disclosing that he had the same variant as his affected children which could explain his own learning difficulties (Figure 3: Excerpt 1). This appeared to be upsetting news for the father who was consoled by his partner in response.

In a different face-to-face consultation between a clinical geneticist and a mother receiving a VUS for their child, the clinician explained that the biggest challenge in ascertaining the pathogenicity of the VUS was that it had been inherited from the mother. The clinician then explained that the mother could be exhibiting a milder form of the suspected condition and proceeded to ask questions about the mother’s health and experiences at school to evidence this (Figure 3: Excerpt 2).

#### 4 Conveying a diagnosis with an uncertain prognosis

**Figure 4:**
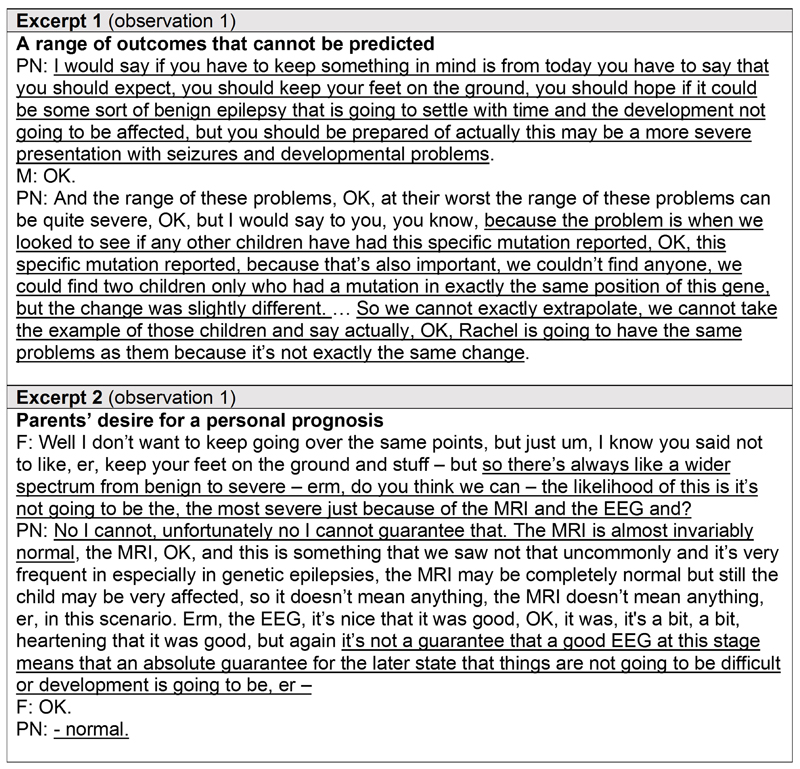
Excerpts from a verbatim transcript of a whole genome sequencing result disclosure consultation demonstrating the challenge of conveying a diagnosis with an uncertain prognosis. Excerpts from a verbatim transcript of a video consultation between a paediatric neurologist and parents receiving a diagnosis of SCN2A channelopathy for their child’s epilepsy from rapid genomic testing, illustrating when a genetic diagnosis did not provide a clearcut prognosis. Pseudonyms are in place of participants’ real names. Key messages are underlined. *PN = paediatric neurologist; M = mother; F = father*.

For several families that received a diagnosis through WGS, limited knowledge about a newly discovered condition or a specific variant meant that future outcomes for the child remained uncertain. The excerpts in Figure 4 were taken from a video consultation between a paediatric neurologist and parents receiving a diagnosis of SCN2A channelopathy for their child’s epilepsy, illustrating a scenario where the uncertainty conflicted with parents’ desire for a personal prognosis for their child. In that consultation, the clinician described the full spectrum of outcomes seen in children with SCN2A channelopathy (involving epilepsy and development) and alerted parents to the low probability that their child would be on the benign end of the spectrum given the absence of a family history of the condition.

The clinician then re-emphasised the indeterminate nature of the child’s outcome as a means of encouraging parents to remain hopeful (Figure 4: Excerpt 1). To justify why it was not possible to predict their child’s future outcomes, the clinician explained that no other children had the exact same variant to inform prognosis (Figure 4: Excerpt 1). The parents contributed little to this information-giving portion of the consultation.

However, approximately halfway into the consultation, when the clinician invited the parents to ask questions, they queried the non-committal prognostication with evidence of what they felt could be signs for a good prognosis, such as a normal MRI and EEG, to which the clinician explained why those were not predictors of outcome and reaffirmed uncertainty (Figure 4: Excerpt 2).

## Discussion

This study highlights some of the empirical challenges in returning WGS results to parents of children with NDDs, which included: 1. explaining a diagnosis when the genotype was established before detailed phenotyping, 2. navigating follow-up for an adult-onset condition identified in childhood, 3. disclosing an unexpected diagnosis for a parent from trio testing, and 4. conveying a diagnosis with an uncertain prognosis.

### Explaining a diagnosis when the genotype was established before detailed phenotyping

Historically, genetic testing has been guided by a specific phenotype, making atypical, heterogenous, and very rare conditions difficult to diagnose. One advantage of WGS is that a wide range of genes linked to a non-specific phenotype can be investigated in a single test. In our study, clinicians often collected details about a child’s phenotype *after* a genetic diagnosis had been made through WGS. We saw that establishing phenotypic compatibility with the diagnosis formed part of the process of informing parents about their child’s result. This involved an exchange of phenotypic information (held by parents) and genotypic information (held by clinicians) to make the relationship between the two visible. This finding aligns with other studies that analysed genetic consultations in the pre-genomic era [26–29], which describe the role of parents in constructing evidence about their child’s problems and participating in diagnostic talk to negotiate a genetic diagnosis during interactions.

A critical difference between the findings of those studies and this study is the stage of the diagnostic journey at which parents were enlisted to provide evidence about their child: prior to a diagnosis as part of the work to identify the genetic cause (those studies) or post-diagnosis to contextualise the genetic cause (this study). Although we do not know what was said or done during pre-test interactions between parents and clinicians in this study, this finding could indicate reduced involvement of parents in the collaborative work required to reach their child’s genetic diagnosis when genomic testing is less reliant on a specific phenotype. A future ethnographic study of genomic testing for paediatric rare disease, including analysis of actual on the ground consultations, could be useful in determining the nature and extent of parents’ participation in the diagnostic process, how this differs across conditions, and if this influences their experience, understanding, and perception of the diagnosis.

### Navigating follow-up for an adult-onset condition identified in childhood

Adult-onset conditions identified in children represent an ethical challenge to genomic practice [30]. Recent guidance has addressed when, and which, incidental findings from genomic testing should be reported to NHS patients or their parents depending on the clinical actionability, penetrance, and classification of the variant [31]. Broader recommendations around genetic testing and counselling for asymptomatic minors include those made by the European Society of Human Genetics [32]. However, less attention has been paid to standardising the logistical follow up of an adult-onset condition identified in childhood. In our study, roles and responsibilities for follow-up were negotiated with the child’s mother and it was ultimately deemed their responsibility to get their child re-referred to clinical genetics once they reached adulthood.

There are several practical and ethical issues to consider when making shared decisions about parental responsibility for follow-up. For example, this relies upon parents *choosing* to re-engage with the service, a choice which may be contingent on their understanding of the incidental finding and its implications. In interviews with parents who received an incidental finding from sequencing in a translational research study [33], those who chose not to pursue recommendations for follow-up were influenced by how important they interpreted the information to be.

Secondly, parents may encounter barriers to getting a referral, which could introduce health disparities if certain groups – such as those already caring for a child with a chronic condition – are less likely to take on those challenges [34]. As an example, in interviews with parents about their experiences of genomic testing in the Australian healthcare system [35], a reported barrier to referral to clinical genetics was that GPs and paediatricians were unsure how to do so.

Finally, in the context of a child with intellectual disability whose future levels of independence may not be clear at the point of decision-making, questions arise around the transfer of responsibility for follow-up should their parent no longer be able and what contingencies may need to be put in place.

To understand what comprises good practice, interviews with parents and clinicians could explore what drives decision-making about logistical follow-up, the reality of implementing those decisions, and the burden of responsibility. In the meantime, parents taking responsibility for follow-up may benefit from written information to recall routes of recontact and referral (which could also support colleagues making the referral) and to reinforce the implications of their child’s incidental finding.

### Disclosing an unexpected diagnosis for a parent from trio testing

A child with a rare disease is more likely to receive a genetic diagnosis from WGS if both of their unaffected parents are included in sequencing for comparison [36]. However, as seen in our study, trio testing may reveal an unexpected disease status in parents if a suggestive phenotype went unrecognised or unreported prior to testing. Such a situation was also described in a study of 16 cases of reverse phenotyping to resolve discrepancies between the segregation of a causative variant identified from exome sequencing and disease status assignment in a family member [37], where phenotypic clarification after testing resulted in seven parents previously considered unaffected being re-evaluated as having the condition.

Thorough phenotypic assessment of parents is important not only to correctly assign disease status for accurate variant interpretation, but also to ensure that before giving consent parents are made aware of the potential information that entering trio testing could reveal about themselves. For example, in interviews with parents about their preferences for incidental findings from their child’s WGS [38], some parents did not want to learn genetic information about themselves uncovered from testing.

An unexpected diagnosis for parents may be less relevant in the context of trio testing for paediatric indications outside of developmental disorders, such as cardiac conditions, where parental phenotypes can be clinically measured and require less subjectivity and sensitivity to define than phenotypes related to cognitive impairment. Further research is needed to understand if clarifying parental phenotypes after trio testing uncovers diagnostic information for parents of children with other rare disease indications, as well as their preferences for disclosure and the effects of this.

### Conveying a diagnosis with an uncertain prognosis

We found that clinicians had open discussions with parents about the extent to which their child’s genetic diagnosis could predict future outcomes. They framed uncertainty as a reason for parents to remain hopeful. This transparent approach to communication aligns with findings from a recent literature review on prognostication in genetic neurodevelopmental conditions [39], which found that parents preferred balanced, strengths-based information that acknowledges uncertainty when talking about their child’s prognosis.

Another key finding of that review was that parents wanted discussions to be tailored to their preferences about the timing, depth, and delivery of prognostic information [39]. In the consultation between a paediatric neurologist and parents of a child diagnosed with SCN2A channelopathy presented in this study, it was apparent that those preferences were not elicited before information delivery. Given that testing had been done to diagnose the child’s epilepsy, but the child was otherwise showing normal signs of development, those parents may not have been prepared to hear about the range, severity, and unpredictability of developmental problems associated with the condition. Talking to parents about their readiness and preferences for prognostic conversations, particularly when outcomes are uncertain, and utilising techniques such as pacing and staging information could help to tailor information to what they are ready to hear [39].

Some parents may be unprepared to receive a genetic diagnosis through WGS that leaves uncertainties around prognosis. For example, in a survey of parents’ knowledge after consenting to WGS through the GMS, a quarter had not understood that WGS may not provide meaningful health information and that there are uncertainties about what a person’s genome can tell them [40]. Exploring parents’ understanding and expectations of WGS during pre-test counselling could help to address misconceptions, foster realistic hopes, and improve preparedness for uncertain results.

In the face of an uncertain trajectory, little is known about if and how clinicians come back to conversations about prognosis over a child’s lifetime. In an analysis of letters returning WGS results to parents of children with intellectual disability [41], clinicians often recommended a future patient review to discuss the child’s condition considering new knowledge that had accrued since the first return of results consultation. Such approaches may be important to help families adjust their perspectives, goals, and plans with evolving knowledge; so too for clinicians to gather information for clinical/research recommendations and case reports/series to potentially reduce uncertainties for future families through knowledge-sharing.

### Implications for practice

Complex, uncertain, and unexpected results arising from genetic and genomic testing are not new issues. Traditionally, these results have been handled and communicated by teams of clinical geneticists and genetic counsellors with expertise in dealing with the psychosocial, family, and ethical issues that can arise. Now that clinicians from mainstream (non-genetics) specialisms can order and return results from certain genomic tests in the NHS, our findings highlight the need to prepare the mainstream workforce for the challenges they may face and effective ways to deal with them. In a survey to evaluate the confidence of paediatricians providing WGS in England, confidence was lowest for discussing complex results with families [42]; our findings support appeals and endeavours to effectively employ genomic counselling skills in mainstream settings, such as through multidisciplinary team meetings and ‘genomic champions’ [43,44], to ensure that the emerging psychosocial, family, and ethical issues raised by genomic testing are identified and appropriately handled as practice evolves [23,45,46].

### Strengths and limitations

The consultations observed in this study captured a range of clinical situations, family structures, and professional roles and experience to reflect diversity in practice. However, several consultations involved clinicians with ‘a lot of experience’ with genomics and parents educated to A-Level or higher (Table 1); different challenges may arise in consultations involving clinicians with less experience or families with lower literacy levels. Future research could further explore results communication by mainstream (non-genetics) clinicians, who may have less experience, to understand challenges encountered and how these are managed. All consultations returned WGS results for NDDs (although disease indication was not specified in recruitment), therefore, our findings may reflect issues specific to NDDs. Since clinicians were responsible for selecting consultations for observation, selection bias cannot be excluded; for example, non-invitation of some parents despite meeting the eligibility criteria could have occurred if clinicians, say, anticipated difficulty during the interaction [47]. Clinician-parent interactions outside of the observed consultation (such as pre-test counselling) were not assessed and may have influenced what was said and done.

## Conclusion

Our study identified and illustrated challenges that emerged when returning results from WGS to parents of children with a NDD, particularly when results were unexpected or uncertain. We demonstrate the need for further study of actual consultations as they unfold in real time to inform guidance on communicating WGS results, ideally across different settings and conditions to understand the similarities and differences in challenges faced and how these are addressed.

## Figures and Tables

**Table 1:** Characteristics of 10 audio-recorded consultations between clinicians and parents to discuss results from whole genome sequencing for their child’s rare disease diagnosis. Demographic data about persons present included where possible.

Case	Patient’s age and gender	Clinical indication for testing	Result type	Mode of appointment	Family member(s) present and their age, gender, and highest qualification	Clinician(s) present and their age, gender, and self-reported experience with genomics (none, some, a lot)
1*	Female, ≤5 years	Unknown†	Diagnosis: SCN2A channelopathy (from rapid genome testing)	Video call	Mother† Father (26-35 years,White, Bachelor’s degree) Paediatric patient	Paediatric neurologist (≥46 years, male) †
2	Male, 11-16 years	Paediatric disorders; Early onset or syndromic epilepsy; Hereditary ataxia with onset in childhood; Childhood onset hereditary spastic paraplegia	No primary finding	Phone call	Mother†	Clinical genetics (female, a lot of experience)†
3	Male, ≤5 years	Unknown†	Diagnosis: Cohen Syndrome	Face-to-face	Mother (36-45 years, Asian British, Master’s degree) Paediatric patient	Clinical geneticist (36-45 years, male, a lot of experience)
4	Female, ≤5 years	Unknown†	Diagnosis: Noonan Syndrome	Phone call	Mother (36-45 years, White, Vocational qualification)	Clinical geneticist (female) †
5*	Female, ≤5 years	Paediatric disorders; Early onset or syndromic epilepsy	VUS in *KDM4B*	Face-to-face	Mother (White, Vocational qualification) Paediatric patient	Clinician geneticist (36-45 years, female, a lot of experience)
6*	Female, ≤5 years	Paediatric disorders	VUS in *TUBB4A* and incidental finding (hereditary breast and ovarian cancer)	Face-to-face	Adoptive mother (≥46 years, Asian, Bachelor’s degree)	Clinical geneticist (≥46 years, female, a lot of experience) Genetic counsellor (26-35 years, female, no experience)
7*	Male, ≤5 years	Intellectual disability	Diagnosis: TLB1XR1-related disorder	Video call	Mother (36-45 years, White, GCSEs) Father (26-35 years, White, GCSEs) Paediatric patient	Clinical geneticist (female)†
8	Female, 11-16 years	Paediatric disorders	Diagnosis: HNRNPU condition	Phone call	Mother (26-35 years, White, A-levels)	Genetic counsellor (26-35, female, a lot of experience)
9*	Female, ≤5 years	Intellectual disability	Diagnosis: SEN8A-related disorder	Face-to-face	Mother† Father† Paediatric patient Older sibling of paediatric patient	Clinical geneticist (36-45 years, male, a lot of experience) Supporting clinician (role unknown)†
10	Female, ≤5 years	Unknown!	Diagnosis: CASK-related disorder	Face-to-face	Foster mother (36-45 years, White) †	Clinical geneticist (36-45 years, female, a lot of experience)

* from observation presented in this paper.

† Incomplete or missing demographic data due to time or logistical constraints to data collection.

## Data Availability

Portions of de-identified transcripts are available upon reasonable request directed to the corresponding author (HE). Full transcripts are not publicly available due to patient confidentially.

## What is already known on this topic

- Despite a growing body of evidence on experiences and outcomes of whole genome sequencing (WGS) in diagnosing rare disease, little is known about the process of communicating WGS results to families in the NHS or how responsive traditional communication and counselling practices are to the shift from genetic to genomic medicine.

## What this study adds

- We identified and examined issues that emerged in interactions between clinicians and parents during consultations to discuss the results of WGS for their child’s neurological or developmental disorder (NDD) and considered the implications for practice.

## How this study might affect research, practice, or policy

- Our study offers insight into the psychosocial, practical, and ethical issues that clinicians may encounter and need to prepare for when returning results from a comparatively genotype-first approach to diagnosing NDD through WGS.
- We demonstrate the nuances of ‘complex’ genomic results, which may require clinicians from mainstream (non-genetics) specialities to seek additional input from genetics specialists to return and manage effectively.
- Future research should continue to record and analyse real consultations in which WGS results are discussed with families, across different settings and conditions. This will help identify emerging challenges and inform strategies to improve communication between clinicians and families.
